# LINC00511 exacerbated T-cell acute lymphoblastic leukemia via miR-195-5p/LRRK1 axis

**DOI:** 10.1042/BSR20193631

**Published:** 2020-05-07

**Authors:** Shengli Li, Wenwen Guo, Huayun Geng, Chao Wang, Shuige Yang, Xinxin Xu

**Affiliations:** 1 Department of Hematology, Jining No.1 People’s Hospital, No. 6 Health Road, Rencheng District, Jining 272100, Shandong, China; 2 Department of Hematology, Dongchangfu People’s Hospital of Liaocheng, 281 Dongguan Street, Dongchangfu District, Liaocheng 252000, Shandong, China; 3 Department of Emergency, Zi Bo Central Hospital, 54 Communist Youth League West Road, Zhangdian District, Zibo 255000, Shandong, China; 4 Department of Hematology, Zi Bo Central Hospital, 54 Communist Youth League West Road, Zhangdian District, Zibo, 255000 Shandong, China

**Keywords:** LINC00511, LRRK1, miR-195-5p, T-cell acute lymphoblastic leukemia

## Abstract

T-cell acute lymphoblastic leukemia (T-ALL) is a malignant disease arising from the abnormal proliferation of T lymphocyte in marrow. Long non-coding RNAs (lncRNAs) are one kind of non-coding RNAs (ncRNAs), which were reported to modulate the initiation or progression of diverse cancers. However, the role of LINC00511 in T-ALL was unknown. To figure out the function and mechanism of LINC00511 in T-ALL, a series of experiments were carried out. Based on the experimental results, we discovered that LINC00511 boosted cell proliferation and invasion, but hindered cell apoptosis in T-ALL cells. Besides, based on bio-informatics tool, miR-195-5p was selected for further exploration. Then, miR-195-5p was validated to bind with LINC00511. Hereafter, *LRRK1* was testified to serve as a target gene of miR-195-5p. At last, rescue assays suggested that *LRRK1* overexpression restored sh-LINC00511#1-mediated effects on cell proliferation and apoptosis. All in all, LINC00511 exacerbated T-ALL progression via miR-195-5p/LRRK1 axis, implying a potential therapeutic clue for the patients with T-ALL.

## Introduction

T-cell acute lymphoblastic leukemia (T-ALL), generally arising from the abnormal proliferation of T lymphocyte in marrow, has been seen as a malignant disease [1,2]. The aberrant lymphocyte proliferation is able to inhibit hematopoietic function. T-ALL is caused by the malignant transformation of T-cell progenitors and occupies approximately 10% of pediatric ALL cases and approximately 25% of adult ALL cases [3,4]. Known factors related to T-ALL are genetic changes, familial inheritance and lionizing radiation, but the pathogenesis of T-ALL remains unclear [5–7]. Hence, it is of great urgency to explore the initiation and progression of T-ALL.

Long non-coding RNAs (lncRNAs) refers to a category of non-coding RNAs (ncRNAs) with no less than 200 nucleotides in length without the capability to encode protein [8,9]. Functionally, lncRNA is identified to frequently participate in the regulation of several cellular processes in diseases or tumors [10]. GAS5 has been proved to regulate cardiac fibroblast activation and fibrosis [11]. In addition, SNHG14 was reported to contribute to cell proliferation of gastric cancer cells through miR-145/SOX9 axis [12]. Recently, long intergenic non-protein coding RNA 511 (LINC00511) was widely reported in multiple tumors. LINC00511 facilitates the breast cancer cell proliferation, invasion and stemness via regulation on miR-185-3p/E2F1/Nanog axis; LINC00511 is induced by SP1 and sponges miR-124-3p to up-regulate *CCND2*, thus accelerating glioma progression; LINC00511 positively regulates *VEGFA* expression through endogenously sponging miR-29b-3p to increase cell proliferation, migration, invasion and endothelial tube formation in pancreatic ductal adenocarcinoma [13–15]. However, the biological function of LINC00511 in T-ALL remains uncharacterized.

Mechanistically, a multitude of researches claimed that lncRNA could function as a competitive endogenous RNA (ceRNA) by sequestering micro RNA (miRNA) to release target gene, and thereby modulate the biological processes in tumors. For instances, MALAT1 accelerates cell proliferation and migration of renal cell carcinoma cells by sponging miR-203 and increasing *BIRC5* expression [16]. SNHG5/miR-26a/SOX2 axis promotes proliferation of chondrocyte in osteoarthritis [17]. Nevertheless, the molecular mechanism of LINC00511 in T-ALL is unknown. In present research, we operated a series of experiments to explore the role of LINC00511 in T-ALL cells.

## Materials and methods

### Tissue samples

The present study was executed from 2013 to 2018, with the ethical approval from the Ethics Committee of Jining No.1 People’s Hospital, and all participants had signed the written informed consent. The blood samples of 35 T-ALL patients and 30 normal controls were collected and instantly maintained in the liquid nitrogen at −80°C.

### Cell lines

Human T-ALL cell lines (HPB-ALL, TALL-1, ALL-SIL, CUTLL1) and peripheral blood mononuclear cells (PBMC) as control were both procured from the American Type Culture Collection (ATCC; Rockville, MD), cultivated in RPMI-1640 medium (Gibco, Grand Island, NY) at 37°C in 95% air/5% CO_2_. Approximately 10% fetal bovine blood (FBS; Gibco) was employed for cell culture.

### Quantitative real-time PCR (qRT-PCR)

The RNeasy/miRNeasy Mini kit (Qiagen, Limburg, The Netherlands) was obtained for extraction of RNA sample from HPB-ALL and TALL-1 cells, followed by reverse-transcription using RevertAid™ First Strand cDNA Synthesis kit (Fermentas, Vilnius, Lithuania). SYBR-Green PCR Master Mix (Applied Biosystems, Foster City, CA) was applied for qPCR on ABI PRISM 7700 Sequence detection system (Applied Biosystems). Calculation for RNA level was performed with 2^−ΔΔCt^ method. The primers of relative genes were provided in Table 1.

**Table 1:** Primers used for PCR

**LINC00511:**
Forward: 5′-CAGGCATGTGGGGCTTTACT-3′;
Reverse: 5′-TCACGTACCTCACCCAACAC-3′;
**miR-195-5p:**
Forward: 5′-TAGCAGCACAGAAATATTGGCGC-3′;
Reverse: 5′-CTCTACAGCTATATTGCCAGCCAC-3′
**miR-625-5p:**
Forward: 5′-AGGGGGAAAGTTCTATAGTCCGC-3′
Reverse: 5′-CTCTACAGCTATATTGCCAGCCAC-3′
**miR-15b-5p:**
Forward: 5′-TAGCAGCACATCATGGTTTACAGC-3′
Reverse: 5′-CTCTACAGCTATATTGCCAGCCAC-3′
**miR-497-5p:**
Forward: 5′-CAGCAGCACACTGTGGTTTG-3′
Reverse: 5′-CTCTACAGCTATATTGCCAGCCAC-3′
**BACE1:**
Forward: 5′-CCTTCATCTATCTGCAAGCCCA-3′
Reverse: 5′-GAGAGTCAAAGAAAGGCTCCAGG-3′
**MYB:**
Forward: 5′-GTCTTCAAAAGCCAGCCAGC-3′
Reverse: 5′-GCTGCATGTGTGGTTCTGTG-3′
**ZBTB34:**
Forward: 5′-CTTGCGGAAAAACCACCCAG-3′
Reverse: 5′-GAGGCCGATGCGTCATTTTC-3′
**AMER1:**
Forward: 5′-GCCTGAGTTACCCTGCCAAT-3′
Reverse: 5′-TTTGGCTTGGGCATAGAGGG-3′
**LRRK1:**
Forward: 5′-CTGCTTTGCAGGCACCAAAA-3′
Reverse: 5′-TTGCTAGTCTTTTAGGCTTTGGAC-3′
**GAPDH:**
Forward: 5′-AATGGGCAGCCGTTAGGAAA-3′
Reverse: 5′-GCGCCCAATACGACCAAATC-3′
**U6:**
Forward: 5′-CTCGCTTCGGCAGCACA-3′
Reverse: 5′-AACGCTTCACGAATTTGCGT-3′

### Plasmid transfection

The confluent T-ALL cell lines at approximately 80% cell density were prepared in 24-well plates and transfected for 48 h by use of Lipofectamine 2000 (Invitrogen, Waltham, MA). The short hairpin RNAs (shRNAs) targeting LINC00511LINC00511 (sh-LINC00511LINC00511#1/2/3) and control (sh-NC), miR-195-5pmiR-195-5p mimics and NC mimics, pcDNA3.1/*LRRK1* and control (pcDNA3.1) were all synthesized at the Shanghai GenePharma (Shanghai, China).

### CCK-8 assay

T-ALL cells in 96-well plates (5 × 10^3^ /well) were prepared for 4 h of incubation with the 10 μl of CCK-8 (Beyotime, Shanghai, China) at 37°C. Absorbance was examined at 450 nm by microplate reader.

### EdU incorporation assay

Transfected T-ALL cells in 96-well plates were used for incubation with 100 μl of EdU incorporation assay kit (Ribobio, Guangzhou China), then with 1× Apollo® 488 fluorescent and DAPI staining after fixation. The EdU-positive cells were observed by fluorescent microscope (Leica, Wetzlar, Germany).

### Flow cytometry for apoptosis

T-ALL cells after transfection were trypsinized and then rinsed in phosphate-buffered saline (PBS). After being treated with Annexin V-FITC Apoptosis Detection Kit (BD Biosciences, San Jose, CA), cells on ice were analyzed through flow cytometry using FACSCalibur (BD Biosciences).

### TUNEL staining

Transfected T-ALL cells were fixed and dyed with 1% formaldehyde and 0.2% Triton X-100 for preparing TUNEL assay. Following mixing with dUTP-end labeling (Clontech, Mountain View, CA) and DAPI solution, TUNEL-positive cells were exposed to fluorescence microscope.

### RNA pull-down assay

RNA pull-down assay was achieved by using Pierce Magnetic RNA-Protein Pull-Down Kit (Thermo Fisher Scientific, Waltham, MA). Protein extracts from HPB-ALL or TALL-1 cells were collected for incubation with 50 pmol of biotinylated RNAs (Bio-miR-195-5p-Wt/Mut and Bio-NC) along with the streptavidin agarose magnetic beads (Thermo Fisher Scientific). The collected complex was examined by qRT-PCR.

### Dual-luciferase reporter assays

The wild-type and mutant miR-195-5p binding sites within LINC00511 sequence or LRRK1 3′-UTR were inserted to pmirGLO vector (Promega, Madison, WI). The reporter vectors LINC00511-WT/Mut and LRRK1-WT/Mut were constructed and then co-incubated with the miR-195-5p mimics or NC mimics into T-ALL cells. Dual-Luciferase Reporter Assay System (Promega) was applied after 48 h for luciferase assay.

### Western blot

The 10% SDS-PAGE was used for the electrophoresis of the extracted protein samples from HPB-ALL and TALL-1 cells. Samples on PVDF membranes were subjected to incubation with primary antibodies against LRRK1 (ab228666; Abcam, Cambridge, MA) and GAPDH (ab9484; Abcam), as well as secondary antibody labeled with HRP. Signals of bands were analyzed by ECL Substrates (Millipore, Bedford, MA).

### Statistical analysis

Each procedure of all assays was repeated independently for more than three times in the present study. Values were shown as mean ± SD (standard deviation). Unpaired *t* test and ANOVA (one- or two-way) were severally utilized for analyzing data of two groups or multiple groups, by use of SPSS 19.0 (SPSS Inc., Chicago, IL). Group difference was significant with the value of *P* < 0.05*.*

## Result

### LINC00511 aggravated T-ALL cell proliferation and inhibiting T-ALL cell apoptosis

**Figure 1:**
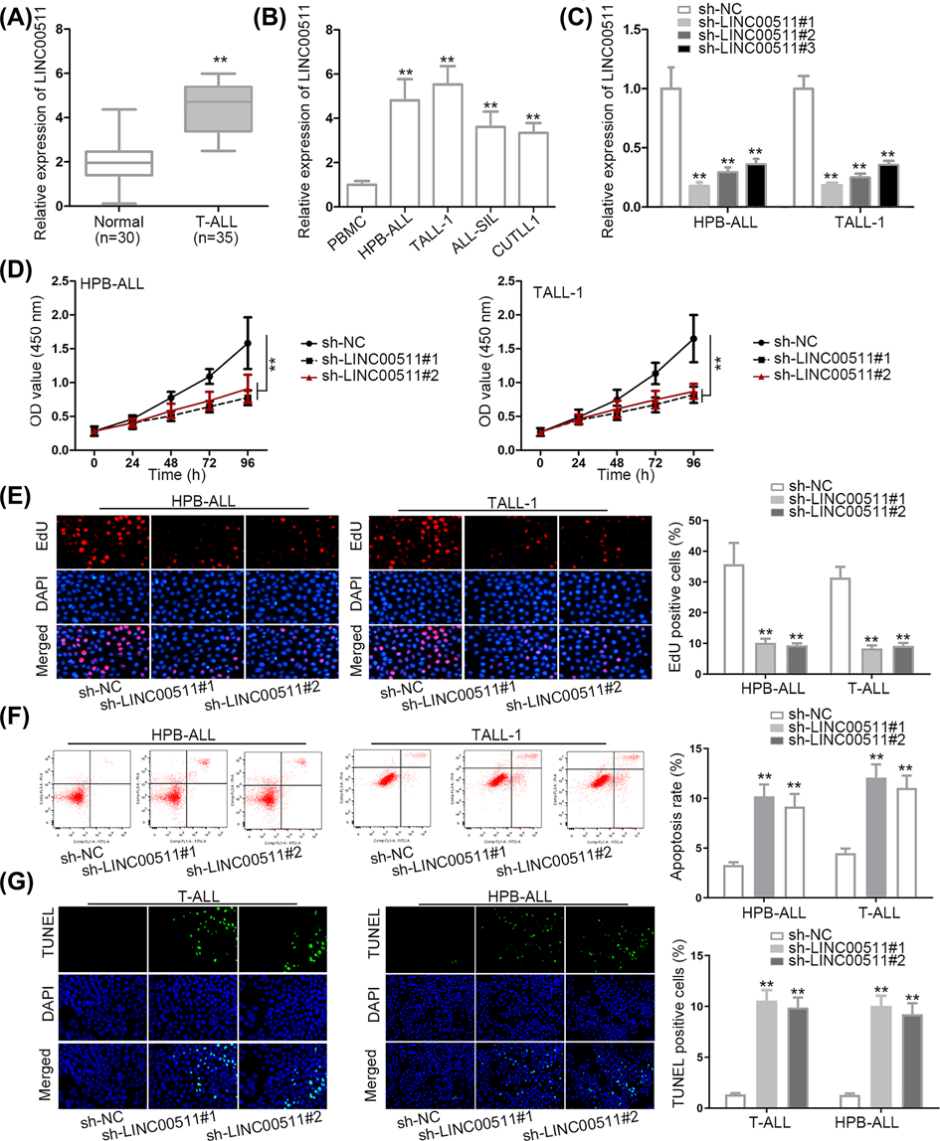
LINC00511 aggravated T-ALL cell proliferation and inhibiting T-ALL cell apoptosis (**A** and **C**) LINC00511 level in blood samples from patients with T-ALL and normal blood samples; LINC00511 level in T-ALL or PBMC cells; the knockdown efficacy of LINC00511 was tested by qRT-PCR assay. (**D** and **E**) Cell proliferation was evaluated by CCK-8 and EdU assays. (**F** and **G**) Cell apoptosis was assessed by TUNEL and flow cytometry assays; **P <0.01.

Numerous studies have identified that LINC00511 promoted the incidence of various cancers. To investigate the specific function of LINC00511 in T-ALL cells, several assays were implemented. qRT-PCR assay indicated that the expression of LINC00511 was up-regulated in blood samples from T-ALL patients than in normal blood samples; also, LINC00511 was up-regulated in T-ALL cells than in peripheral blood mononuclear cells (PBMC) (Figure 1A,B). Then, LINC00511 expression was dramatically decreased by the transfection of sh-LINC00511#1/2/3 into HPB-ALL and TALL-1 cells (Figure 1C). Afterward, sh-LINC00511#1/2 was chosen for loss-of-function assay for their better knockdown efficacy. As shown in CCK-8 assay, LINC00511 knockdown caused an evident reduction on cell proliferation (Figure 1D). Likewise, the EdU positive cells were also reduced by LINC00511 suppression (Figure 1E). On the contrary, flow cytometry and TUNEL assays illustrated that cell apoptosis was boosted by transfecting sh-LINC00511#1/2 into HPB-ALL and TALL-1 cells (Figure 1F,G). Taken together, LINC00511 aggravated T-ALL cell proliferation and inhibited apoptosis.

### MiR-195-5p, sponged by LINC00511, alleviated T-ALL by weakening proliferation and accelerating apoptosis

**Figure 2:**
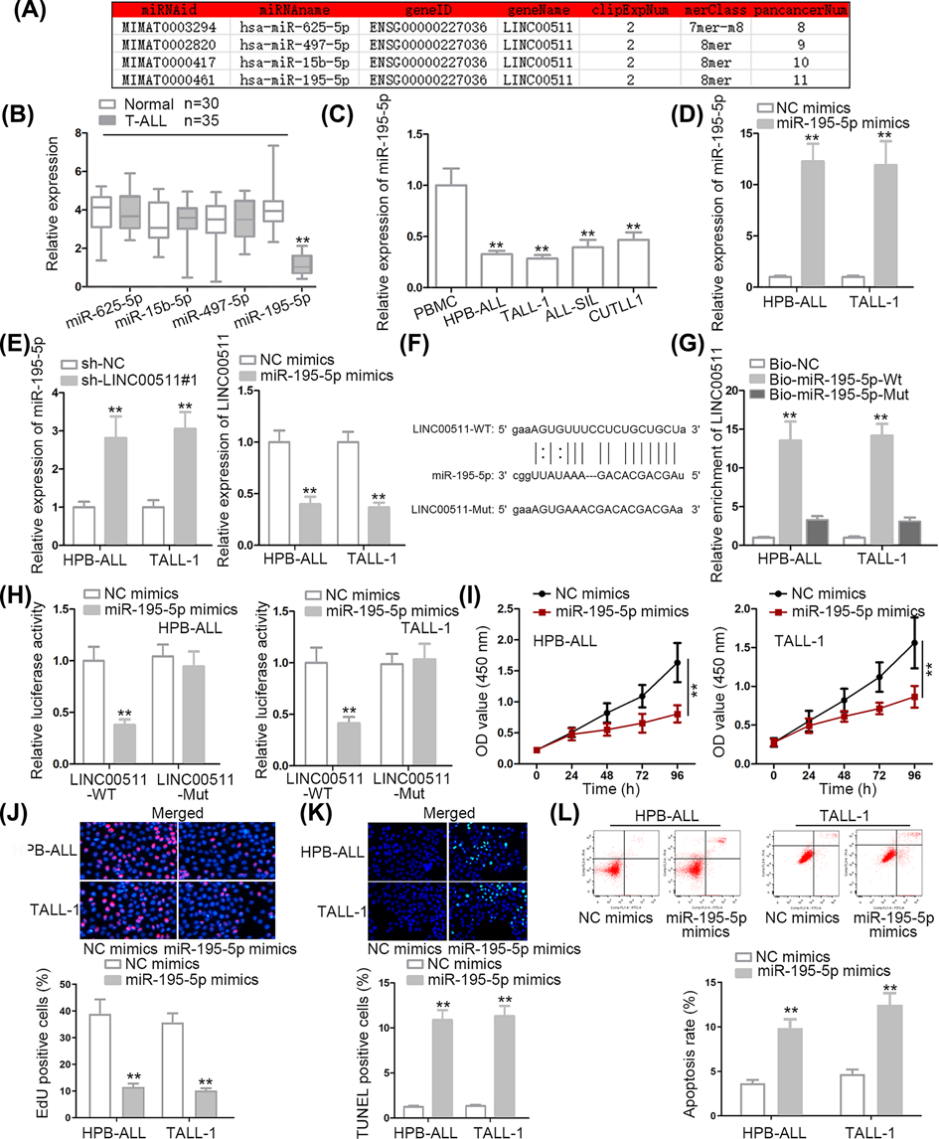
MiR-195-5p, sponged by LINC00511, alleviated T-ALL by weakening proliferation and accelerating apoptosis (**A**) The potential miRNAs were predicted by starBase. (**B**) The predicted miRNAs levels were checked by qRT-PCR assay. (**C** and **D**) The expression level and overexpression efficiency of miR-195-5p in T-ALL cells were explored by qRT-PCR assay. (**E**) The interaction between LINC00511 and miR-195-5p was determined by qRT-PCR assay. (**F**) The binding sequences between LINC00511 and miR-195-5p were predicted by starBase. LINC00511-WT referred to mutant binding sequences of LINC00511. (**G** and **H**) RNA pull-down and luciferase reporter assays verified the combination between LINC00511 and miR-195-5p. (**I** and **J**) CCK-8 and EdU assays were employed to measure cell proliferation. (**K** and **L**) TUNEL and flow cytometry assays were operated to investigate cell apoptosis; **P <0.01.

A multitude of studies have proved that lncRNA acted as a ceRNA to exert an influence on target gene expression through sponging miRNAs [17,18]. Thus, we hypothesized that LINC00511 also acted in this way in T-ALL cells. According to starBase (screening conditions: medium stringency of CLIP Data and eight cancer types of Pan-Cancer) (http://starbase.sysu.edu.cn), four miRNAs were chosen for further exploration (Figure 2A). As shown in Figure 2B, compared with normal bloods, only miR-195-5p was down-regulated in T-ALL blood samples whereas other miRNAs displayed no differences. Similarly, T-ALL cells also presented lower miR-195-5p expression (Figure 2C). Afterward, miR-195-5p expression was prominently increased by transfection of miR-195-5p mimics (Figure 2D). On the top of that, LINC00511 inhibition increased miR-195-5p levels whilst miR-195-5p amplification decreased LINC00511 levels (Figure 2E). To evaluate the binding capacity between LINC00511 and miR-195-5p, the binding sequences between LINC00511 and miR-195-5p were predicted; also, the mutated binding sequences of LINC00511 were presented (Figure 2F). RNA pull-down assay demonstrated that LINC00511 was significantly pulled down by Bio-miR-195-5p wild-type group (Figure 2G). Luciferase reporter assay disclosed that the transfection of miR-195-5p mimics triggered a decline of luciferase activity of LINC00511-WT vector and no alteration was noticed in LINC00511-Mut vector (Figure 2H). To explore the role of miR-195-5p in T-ALL, the gain-of-function assays were carried out. CCK-8 and EdU assays revealed that cell proliferation was distinctly limited by miR-195-5p amplification (Figure 2I,J). Inversely, TUNEL and flow cytometry assays delineated that cell apoptosis was conspicuously enhanced by up-regulation of miR-195-5p (Figure 2K,L). In conclusion, miR-195-5p was sponged by LINC00511, weakening proliferation and accelerating apoptosis in T-ALL cells.

### *LRRK1* was targeted by miR-195-5p in T-ALL cells

**Figure 3:**
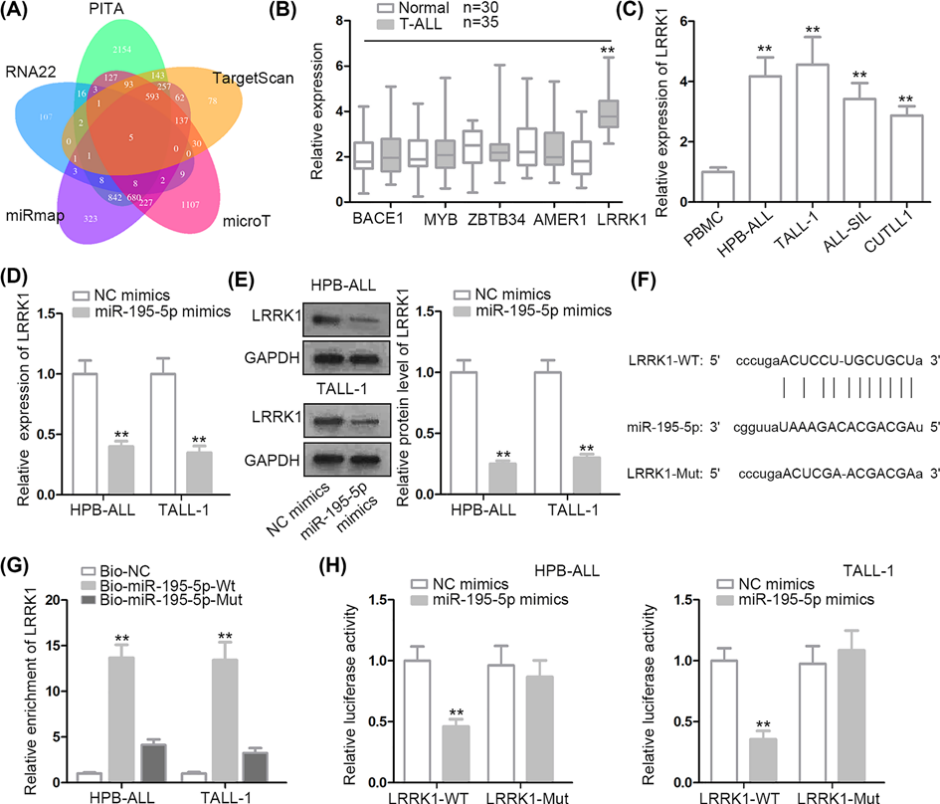
*LRRK1* was targeted by miR-195-5p in T-ALL cells (**A**) Venn diagram presented the number of target genes of miR-195-5p. (**B**) The potential target genes expression in T-ALL blood samples or normal blood samples was examined by qRT-PCR assay. (**C**) *LRRK1* levels in T-ALL cells and PBMC cells were detected by qRT-PCR assay. (**D** and **E**) The mRNA and protein levels of *LRRK1* in the context of miR-195-5p overexpression were respectively monitored by qRT-PCR and Western blot assays. (**F**) The binding sequences between LRRK1 and miR-195-5p were predicted by starBase. LRRK1-WT referred to mutant binding sequences of LRRK1. (**G** and **H**) The binding capacity between LRRK1 and miR-195-5p was validated by RNA pull down and luciferase reporter assays; **P <0.01.

Next, the downstream targets of miR-195-5p were searched by bioinformatics analysis. Venn diagram depicted that five target genes containing the binding sequences of miR-195-5p were screened out based on five programs (Figure 3A). Subsequently, qRT-PCR uncovered that *LRRK1* was up-regulated in T-ALL blood samples while other mRNAs presented no differential expression (Figure 3B). By the same token, T-ALL cells exhibited higher *LRRK1* level than PBMC cells (Figure 3C). Then, miR-195-5p amplification obviously attenuated mRNA and protein levels of *LRRK1* (Figure 3D,E). The binding sequences of miR-195-5p and LRRK1 were predicted by starBase and shown in Figure 3F. RNA pull-down assay unveiled that LRRK1 was abundantly enriched in Bio-miR-195-5p-Wt group (Figure 3G). Additionally, luciferase assay revealed that the luciferase activity of pmirGLO-LRRK1-WT was repressed by miR-195-5p augmentation, and the luciferase activity of pmirGLO-LRRK1-Mut was not altered in the same condition (Figure 3H). Collectively, *LRRK1* was targeted by miR-195-5p in T-ALL cells.

### LINC00511 exacerbated T-ALL by up-regulating *LRRK1*

**Figure 4:**
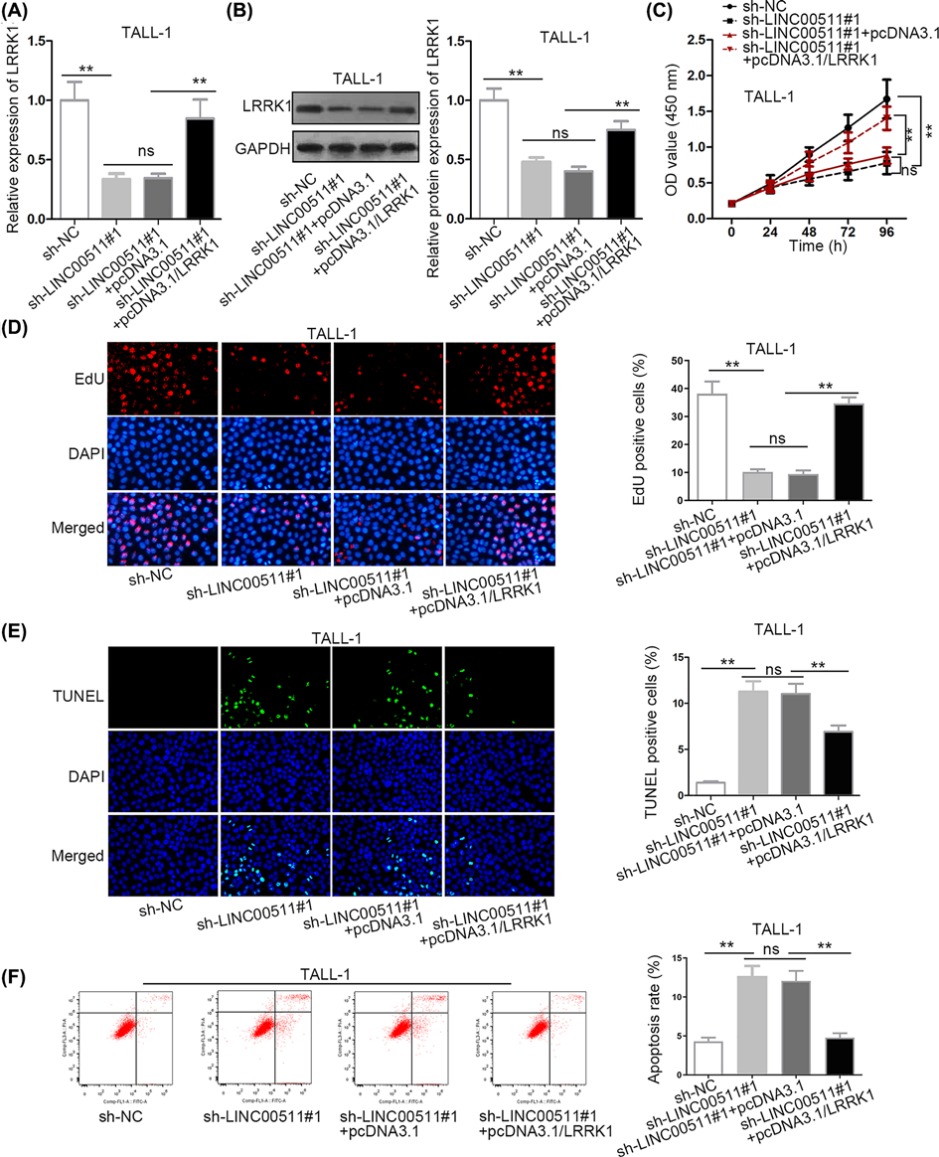
LINC00511 exacerbated T-ALL by up-regulating *LRRK1* (**A** and **B**) The detection of mRNA and protein levels of *LRRK1* were respectively conducted in qRT-PCR and Western blot assays. (**C** and **D**) The measurement of cell proliferation was implemented in CCK-8 and EdU assays. (**E** and **F**) The evaluation of cell apoptosis was operated in TUNEL and flow cytometry assays; **P <0.01; *“*ns” indicates no significance.

*LRRK1* has been reported to regulate the proliferation of B-cells [19]. To confirm that LINC00511 promoted T-ALL development by regulating *LRRK1*, rescue assays were carried out. To begin with, the inhibitory effect of down-regulated LINC00511 on the mRNA and protein levels of *LRRK1* was restored by transfection of pcDNA3.1/*LRRK1* into TALL-1 cells (Figure 4A,B). Furthermore, the augmentation of *LRRK1* recovered the inhibitive influence of silenced LINC00511 on the proliferative ability of TALL-1 cells (Figure 4C,D). Over and above that, the promoting impact of LINC00511 depletion on cell apoptosis was offset by *LRRK1* overexpression (Figure 4E,F). All the experimental result led to a conclusion that LINC00511 exacerbated T-ALL cell proliferation and inhibiting apoptosis by up-regulating *LRRK1*.

## Discussion

T-ALL has been considered to be a leading cause of cancer-related death in children, and thousands of children were newly diagnosed with T-ALL in China [20–22]. The typical symptoms of T-ALL were anemia, fever or arthralgia [23,24]. The prognosis of T-ALL has been improved by adopting novel chemotherapy approaches in recent years [25,26]. Yet, the molecular marker for diagnosis or therapy of T-ALL deserved a further investigation.

Functionally, lncRNA was supposed to play an irreplaceable role in the modulation of cellular processes [27]. Furthermore, the dysregulation of lncRNAs has been confirmed to be closely associated with the occurrence or development of cancers [28]. LncRNA PVT1 was identified to modulate chondrocyte apoptosis in osteoarthritis by decreasing miR-488-3p expression [29]. The up-regulated HOTAIR promotes cell proliferation of prostate cancer cells [30]. Present study uncovered that LINC00511 was up-regulated in T-ALL bloods (or cells) compared with normal bloods (or PBMC). Inhibition of LINC00511 hindered cell proliferation but enhanced cell apoptosis of T-ALL cells.

In mechanism, emerging evidences have proposed a ceRNA pattern in which lncRNA was able to up-regulate target gene expression by binding with the MREs (microRNA response elements) of miRNAs [31]. For example, SNHG16 competes with E2F5 to bind with miR-98, thus promoting breast cancer cell migration [32]. Long non-coding RNA OIP5-AS1 enhances *Bcl-2* expression by reducing miR-448 expression in lung adenocarcinoma [18]. Based on this theory, we conjectured that LINC00511 also functioned in ceRNA pattern. Thus, miR-195-5p was screened out based on the prediction of starBase. Besides, miR-195-5p was down-regulated in T-ALL bloods and cells. Then, the combination between LINC00511 and miR-195-5p was confirmed. Hereafter, we explored the function of miR-195-5p in T-ALL, and we discovered that miR-195-5p overexpression prevented cell proliferation and accelerated cell apoptosis. Moreover, leucine-rich repeat kinase 1 (*LRRK1*), which has been reported to regulate the proliferation of B cells before, was selected as a target gene of miR-195-5p [19]. In our exploration, *LRRK1* displayed higher expression in T-ALL bloods (or cells) than in control bloods (or PBMC cells). Moreover, the binding capacity between miR-195-5p and LRRK1 was validated. Last but not least, rescue assays demonstrated that *LRRK1* overexpression restored sh-LINC00511#1-mediated effects on cell proliferation and apoptosis.

To conclude, we testified that LINC00511 exacerbated T-ALL cell proliferation and inhibited cell apoptosis via miR-195-5p/LRRK1 axis, which implies a potential biomarker of T-ALL treatment. However, the pathology of T-ALL was complicated and this research only focused on the regulatory mechanism of LINC00511. Other mechanisms remained to be investigated in the future.

## Abbreviations

ceRNA: competitive endogenous RNA
lncRNA: long non-coding RNA
miRNA: micro RNA
ncRNA: non-coding RNA
PBMC: peripheral blood mononuclear cells
T-ALL: T-cell acute lymphoblastic leukemia
